# High Quality Performance of Novel Immunoassays for the Sensitive Quantification of Soluble PD-1, PD-L1 and PD-L2 in Blood

**DOI:** 10.3390/biomedicines10102405

**Published:** 2022-09-26

**Authors:** Kimberly Krueger, Zsuzsanna Mayer, Miriam Gerckens, Stefan Boeck, Peter Luppa, Stefan Holdenrieder

**Affiliations:** 1 German Heart Center Munich, Munich Biomarker Research Center, Institute of Laboratory Medicine, Clinics at the Technical University Munich, 80636 Munich, Germany; 2 Department of Internal Medicine III, University Hospital Munich-Grosshadern, 81377 Munich, Germany; 3 Department of Clinical Chemistry, University Hospital of the Technical University Munich, 81675 Munich, Germany

**Keywords:** PD-1, PD-L1, PD-L2, analytical validation, ELISA

## Abstract

Programmed death-1 receptor PD-1(CD279) and its corresponding ligands PD-L1(CD274, B7-H1) and PD-L2(CD273, B7-DC) play important roles in physiological immune tolerance and for immune escape in cancer disease. Hence, the establishment and analytical validation of a novel enzyme-linked immunosorbent assay (ELISA) to measure soluble PD-1, PD-L1 and PD-L2 in blood samples according to high quality standards is required. Antibody pairs were used to establish novel highly sensitive ELISAs for all three markers on an open electrochemiluminescence Quickplex platform. Analytical validation comprised intra- and interassay imprecision, limit of quantification, dilution linearity, material comparison and analytical selectivity testing. The methods demonstrated a broad dynamic range and precise measurements down to the pg/mL range. The coefficient of variation (CV) during the intra-assay imprecision measurements with three patient pools did not exceed 10% for all three assays (PD-1: 6.4%, 6.5%, 7.8%, PD-L1: 7.1%, 4.2%, 6.8%; PD-L2: 4.5%, 10.0%, 9.9%). Dilution linearity experiments in both buffer and heparin plasma displayed good linearity. Selectivity was shown for each marker in titration cross-reactivity experiments up to concentrations of at least 15 ng/mL of these, possibly confounding other markers. Soluble PD-1, PD-L1 and PD-L2 can be measured highly sensitively in serum and plasma and can safely be applied to clinical study settings.

## 1. Introduction

Programmed death proteins PD-1, PD-L1 and PD-L2 participate in an important immune regulatory signaling pathway. PD-1 (32 kDa) is a transmembrane receptor encoded in the Pdcd1 gene. Its highly glycolsylated profile is an important pattern for ligand interaction [1,2]. PD-L1 (33 kDa) and PD-L2 (31 kDA) serve as PD-1’s corresponding ligands and are encoded by the *Cd274* gene and *Pdcd1lg2* gene [1]. Receptors and both ligands have been described to be present on immune cells as well as soluble markers in blood plasma and serum [1,3]. PD-1 is expressed on the surface of activated T-cells, B-cells, myeloid cells and macrophages [3,4,5,6]. The ligands are found on antigen-presenting cells and dendritic cells as well as a variety of other hematopoetic and non-hematopoetic cells [7,8]. Interaction of receptor and ligand downregulates T-cell activity and as such physiologically serves as an immune tolerance mechanism [1,9]. Numerous tumor cells have been shown to use the expression of ligand PD-L1 to evade elimination by the immune system [1,10,11]. The understanding of this interaction has led to the development of immune checkpoint inhibitors (ICI) with pembrolizumab representing the first approved PD-1 inhibitor. The experienced efficacy and safety profile marked the starting point of the still ongoing development of various PD-1 and programmed death-ligand 1 (PD-L1) inhibitors by different companies.

Today, diverse ICIs are listed in therapy guidelines of different solid tumors including melanoma and non-small cell lung cancer (NSCLC) [12]. Their application resulted in a longer progression-free period and overall survival and improved patients’ quality of life [13,14]. Currently, combination therapies are assessed [15].

ICI medication is costly and bears the risk for severe side effects, while only between 10 and 40% of the patients benefit from the treatment [16,17]. Consequently, there is a strong need for inexpensive and reliable patient stratification and monitoring of efficacy in vivo. One of the currently available methods is immunohistochemistry (IHC) staining based on anti-PD-L1 antibodies. The limitations of IHC are widely discussed in the literature and foster the research on other biomarkers, such as tumor mutational burden (TMB), tumor-infiltrating lymphocytes (TILs), immune score and others [17,18,19,20,21]. Limitations of this methods are the incorporation of expensive sequencing data and the lack of capturing tumor heterogeneity and longitudinal surveillance by using biopsy material. The aim of this work is to establish and validate a blood-based ELISA for the sensitive quantification of soluble biomarkers PD-1, PD-L1 and PD-L2. This approach will be able to combine inexpensive measurements on easily accessible sample material with the ability for longitudinal monitoring and individual in vivo efficacy testing, thereby improving oncoimmunology research and sensitive monitoring of anti-cancer immunotherapy.

## 2. Materials and Methods

The methods for all three programmed death protein markers (PD markers) were developed as ELISAs using monoclonal antibodies and recombinant protein standards (R&D Systems, Inc., Minneapolis, MN, USA). The assays were established as chemiluminescence detection methods on the MESO QuickPlex SQ 120 instrument (Meso Scale Discovery, LLC., Rockville, MD, USA), which provided an open platform for self-developed ELISAs. QUICKPLEX^®^96 well plates, SULFO-TAG Streptavidin and MSD GOLD Read Buffer were purchased from the manufacturer (Meso Scale Discovery, LLC., Rockville, MD, USA).

All assays were performed following the standard procedure. The carbon surface of the plate was coated with the capture antibody overnight at 2–8 °C. A blocking step with Albumin Fraction V (Carl Roth GmbH+Co.KG, Karlsruhe, Germany) reduced non-specific sample binding. In the next step, the standards, controls and samples were pipetted on the plate, followed by the biotin-coupled detection antibody. Finally, the streptavidin-coupled detection reagent was incubated. All incubation steps were followed by a three-times washing step using 0,05% TWEEN^®^20 in PBS (TWEEN^®^20, Merck KGaA, Darmstadt, Germany; ROTI^®^-CELL 10x PBS, Carl Roth GmbH+Co.KG, Karlsruhe, Germany). After application of the read buffer, the plate was measured within fifteen minutes on the MESO QuickPlex SQ 120. Data procession and analysis was performed with the supplied Discovery Workbench 4.0.12 (LSR_4_0_12) (Meso Scale Discovery, LLC., Rockville, MD, USA).

The established workflow comprised three main parts: (i) development and optimization of the assays, subsequently followed by standardization and introduction of appropriate quality controls; (ii) comprehensive analytical validation consisting of experiments for general assay settings, matrix effects, imprecision, dilution linearity, assay characteristics and selectivity; (iii) establishment of a workflow for a validated standard operating procedure (SOP) by combining and adapting different recommendations and guidelines.

### 2.1. Assay Development and Optimization

All three assays were based on antibodies and standards purchased from DuoSet^®^ Development Systems (Human PD-1 DuoSet^®^ ELISA: DY1086/ Human PD-L1 DuoSet^®^ ELISA: DY156/ Human PD-L2/B7-DC DuoSet^®^ ELISA: DY1224, R&D Systems, Inc., Minneapolis, MN, USA). The standard curves each consisted of a seven-point serial dilution of the recombinant protein. The highest standard equaled 30 ng/mL. The following six standards were prepared by a 1:4 dilution from the previous one until a concentration of 0.0073 ng/mL was reached. The eighth standard consisted of buffer and served as the blank value. The resulting calibration curves were calculated by fitting the signals from the standards to a 4-parameter logistic (or sigmoidal dose-response) model with a 1/Y2 weighting.

Best antibody combinations and concentrations were defined by so-called chessboard titration experiments of standard curves. Therefore, a grid was constructed to compare two capture antibody concentrations with three detection antibody concentrations (Table S1, Figure S1). The most suitable concentrations were identified by signal-to-noise ratio calculation. Signal optimization was performed by the use of other diluents than the reagent diluent on the standard curve and the detection antibody (Diluent 2/3/43, Meso Scale Discovery, LLC., Rockville, MD, USA). For PD-L1, R&D Systems recommended an addition of 2% heat inactivated normal goat serum (NGS) to the detection antibody diluent, which was tested, too. Additionally, both proffered plate types from Meso Scale Discovery (MSD), Standard plate and High-Bind plate, were compared. The standard plate has a hydrophobic surface, meanwhile, the High-Bind plate presents with a more hydrophilic surface.

### 2.2. Quality Control

To maintain constant quality control (QC), a set of lab-produced in-house controls were included in all plates. These were prepared from pooled patient serum samples in high, medium or low marker levels. To maintain high quality controls, the pools were aliquoted in multiple vials and stored at −80 °C. Endogenous levels of PD-L1 were usually very low, so two of the respective pools were spiked to a high and medium concentration with recombinant protein. These pools served also as experimental material in the course of the validation. The validation experiments were performed according to guidelines on biomarker assay validation [22,23].

### 2.3. Imprecision

For the intra-assay imprecision, three plasma pools with a high, medium and low value of the corresponding biomarker were chosen. This enabled a good coverage of the whole measuring range. Samples were measured in replicates of eleven to fifteen wells on the same plate. The inter-assay imprecision part consisted of a repetitive measurement of two sample pools on different plates. Measurements on up to 14 plates, which covered up to seven days, were included in the analysis.

### 2.4. Dilution Linearity

Primary experiments were performed in assay buffer and artificial matrices (SigMatrix (Serum Diluent, Merck KGaA, Darmstadt, Germany); SeraSubTM (CST Technologies, Inc., New York City, NY, USA)). Diluent 2 (Meso Scale Discovery, LLC., Rockville, MD, USA) for PD-1 and PD-L2 and PBS with 1% BSA for PD-L1 were used. In the second set of experiments, heparin plasma from up to eight donors was used. Probes were used native or spiked to a concentration of at least 10 ng/mL with recombinant protein from R&D Systems (R&D Systems, Inc., Minneapolis, MN, USA). After that, a serial 1:1 dilution row was prepared including dilutions 1:2; 1:4; 1:8 and, if applicable, 1:16 in the respective assay buffer or plasma.

### 2.5. Assay Measuring Range

The limit of detection (LOD) was based on the background signals. Mean and standard deviation both were calculated using 23 or more blank values from different days. The standard deviation was multiplied times 2.5 and added on the mean value to create the LOD [24,25]. The lower and upper limits of quantification (LLOQ; ULOQ) define the range in which the assay provides valid concentrations. Following this approach, for each standard, nine or more standard curves were compared regarding recovery and coefficient of variation (CV). The criteria for LLOQ and ULOQ requested a maximum CV of 20%, meanwhile, the recovery had to be within 80 to 120%.

### 2.6. Selectivity

Selectivity, as one of the crucial assay performance factors, was tested in several experiments. In the first step, standard curve dilutions of the corresponding other components (e.g., PD-L1 or PD-L2) were tested on the target antibody pair (e.g., PD-1). Next, a serial dilution was mixed for each potential disturbing component on its own (e.g., PD-L1 or PD-L2) and a combination of both (e.g., PD-L1 and PD-L2). As spike matrices, two different concentrations of the recombinant protein in buffer (standard 3 and 5) and two patient heparin samples were prepared.

### 2.7. Matrix Effects

Matrix effects were tested on four different sample types: serum, heparin plasma, EDTA plasma and citrate plasma (S-Monovette^®^ 5.5 mL Z, S-Monovette^®^ 5.5 mL LH, S-Monovette^®^ 9 mL K3E, S-Monovette^®^ 5 mL 9NC; all: SARSTEDT AG&Co.KG, Nürmbrecht, Germany). Thereby, recovery of marker levels obtained in heparin, EDTA and citrate plasma was calculated against concentrations in serum. Five patients were included in this pilot study, each of whom contributed blood in all four aforementioned tubes for direct comparison.

### 2.8. Healthy Subjects

PD-1, PD-L1 and PD-L2 concentrations were measured in a cohort of 136 healthy male subjects (age: 32–71) applying the described assays.

### 2.9. Blood Sample Preparation

Whole blood was centrifuged for 10 min at 3000 rcf (3000 g) for plasma generation. The matrix comparison experiment included four matrices, serum, heparin plasma, EDTA plasma and citrate plasma (S-Monovette^®^ 5.5 mL Z, S-Monovette^®^ 5.5 mL LH, S-Monovette^®^ 9 mL K3E, S-Monovette^®^ 5 mL 9NC; all: SARSTEDT AG&Co.KG, Nürmbrecht, Germany), whereas all experiments were conducted in heparin plasma. Plasma samples were aliquoted and stored at −80 °C until analysis.

The majority of experiments were conducted in anonymized residual research samples. Samples for the matrix comparison experiment (maximum 25 mL) were collected as part of quality control for biobanked blood samples. Blood samples were taken from patients of the Department of Cardiology during routine venipuncture or from healthy individuals after informed consent for blood collection for the Cardiovascular Biobank of the German Heart Centre Munich was obtained. The blood collection for biobanking was approved by the Ethics Commission of the Technical University Munich (Nr. 5943/13; 16 October 2013).

### 2.10. Statistics and Data Interpretation

Data analysis was performed using Microsoft Excel 2010. Basic statistic tools comprised of calculation of mean, standard deviation, recovery and coefficient of variation (CV). The requested range of recoveries was between 80% and 120%. In general, the CV should not exceed ±20%, though it was narrowed to 10% for intra-assay imprecision data. For the analysis of dilution linearity, the undiluted sample value was set to 100%. Following dilution results were calculated as the percentage of previous dilution (target = 50%), which allowed a direct assessment of the quality of each dilution step. All obtained results were depicted as bar or line charts. Whenever applicable, error bars showing the CV were incorporated. All concentrations are provided in ng/mL, which equals 10–9 kg/L to ensure ease of readability.

## 3. Results

### 3.1. Assay Development and Optimization

For PD-1, the highest signal-to-noise ratios were calculated for the combination of 2 µg/mL capture antibody with 400 ng/mL detection antibody. The use of MSD Diluent 2 for standard curve dilution further decreased the blank signal and, therefore, optimized signal-to-noise ratios.

For PD-L1, the concentrations of 4 µg/mL for the capture antibody and 100 ng/mL for the detection antibody were confirmed to be used in future experiments. The addition of normal goat serum (NGS) to the detection antibody diluent caused a considerable drop in sample values, whereas MSD Diluent 3 decreased blank signals. Consequently, MSD Diluent 3 was used instead of the NGS-containing buffer for detection antibody dilution.

Finally, for PD-L2, the combination of 2 µg/mL capture antibody and 200 ng/mL detection antibody provided the best results. Similar to the PD-1 results, the use of MSD Diluent 2 for standard curve dilution decreased blank signals, improving overall assay sensitivity.

**Figure 1:**
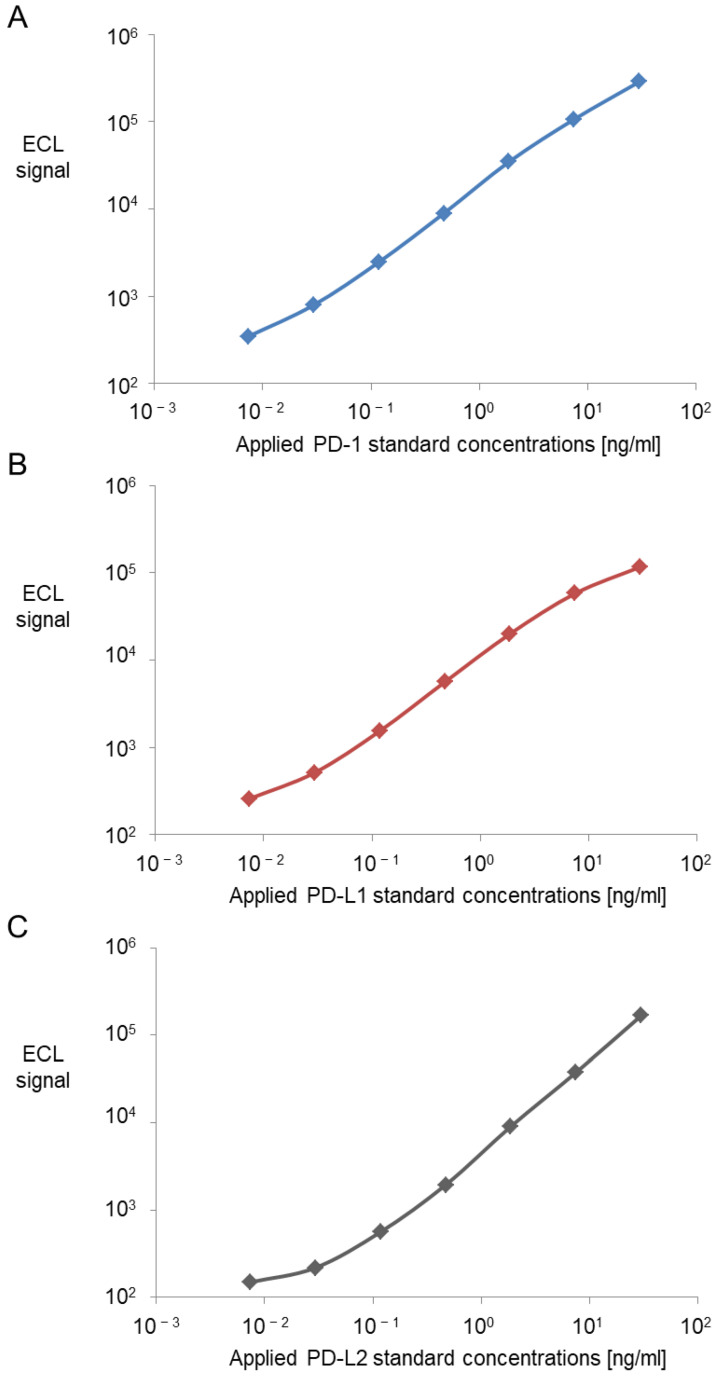
Standard curves of the assays for PD-1 (**A**), PD-L1 (**B**) and PD-L2 (**C**). Measured signals are plotted over a serial dilution of recombinant protein for PD-1 (**A**), PD-L1 (**B**) and PD-L2 (**C**). These serve as standard curves to quantify protein concentrations in following experiments. The calibration curves used to calculate antibody concentrations are established by fitting the signals from the calibrators to a 4-parameter logistic (or sigmoidal dose-response) model with a 1/Y2 weighting. ECL: Electrochemiluminescence.

All three assays worked with the same standard curve scheme. The standards were made of a 1:4 dilution covering the range between 30 ng/mL and 0.0073 ng/mL (Figure 1). The “standard plate” showed a wider spread of signal to noise ratios for all three biomarkers with an n-fold increase of three for PD-1, three for PD-L1 and two for PD-L2 (Table S2). Final determined assay conditions can be found in the Supplementary Materials (Table S3).

### 3.2. Imprecision

The observed range of the coefficient of variation in the intra-assay imprecision experiment was 6.4 to 7.8% for PD-1, 3.8 to 7.1% for PD-L1 and 4.5 to 10.0% for PD-L2 (Table 1). A plate drift of results was not detected in either direction.

The reported changes in inter-assay imprecision experiments ranged from 12.7 to 13.3% (PD-1), 12.5 to 15.5% (PD-L1) and 13.2 to 23.4% (PD-L2) (Table 2).

### 3.3. Dilution Linearity

Linearity of dilution was proven for buffer and artificial matrices and for 1:4 dilutions onwards in the included blood-based matrices (Figure S2). Obtained recoveries for the first dilution step (1:2) in buffer and the two artificial matrices SigMatrix and SeraSub™ started with 36–50% for PD-1 (52–58% for PD-L1 and 43–46% for PD-L2) and followed the aforementioned target values in the latter dilution steps. The plasma experiments for PD-1 and PD-L2 showed higher variation in recovery in the first dilution step (1:2) for the native samples, but not in the spiked samples. Further steps proved a linear decline of the sample value according to the applied dilution indifferent from the used diluent in PD-1 and the spiked samples for PD-L2. Spiked PD-L1 samples resulted in linear dilution results from the first step on. Absolute concentrations and percentages are listed in the Supplementary Materials (Table S4, Figures S3 and S4).

### 3.4. Assay Measurement Range

The LOD was calculated by mean background signals. The LLOQs and ULOQs were calculated using up to 14 standard curve results. Obtained coefficients of variation, calculated from the mean recalculated concentration, showed that the quantification range encompasses the whole standard curve as the lower limit equals the lowest (0.0073 ng/mL) and the upper limit the highest standard (30 ng/mL), respectively (Table 3).

### 3.5. Selectivity

The antibodies used in the PD-1 assay did not bind the recombinant proteins PD-L1 and PD-L2 and vice versa. The signals in those wells equaled the blank signal (Figure S5).

Regarding the PD-1 assay, all results with PD-L1 spikes were located within the predefined ±20% frame. PD-L2 concentrations of 60 ng/mL exceed this predefined range and conclusively the combination of both also does. In the PD-L1 assay, concentrations of 60 ng/mL of either PD-1 or PD-L2 changed the recovery by more than 20%, meanwhile, all the other concentrations did not influence the results. The PD-L2 assay was not influenced by any tested PD-L1 concentration. PD-1 and consequently also the combination of PD-1 and PD-L1 showed decreases of more than 20% in the recovery for the highest concentration of 60 ng/mL only (Figure S6).

**Figure 2:**
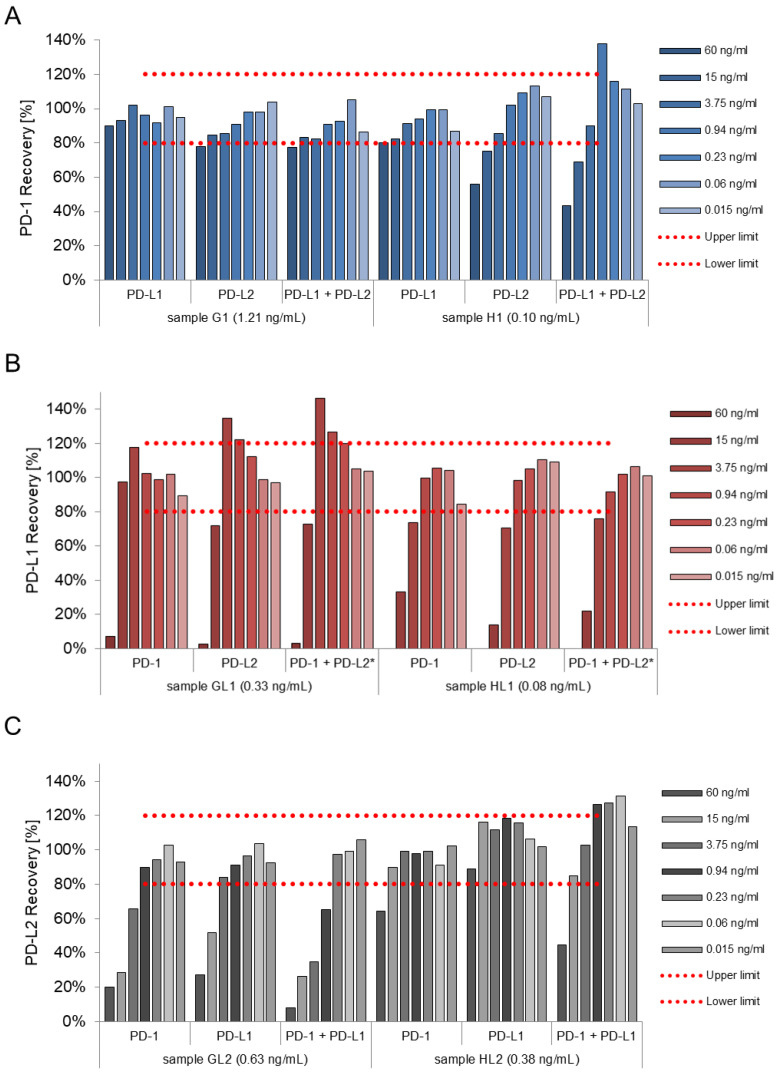
Percentage results of selectivity experiment in heparin plasma for PD-1 (**A**), PD-L1 (**B**) and PD-L2 (**C**). * A starting concentration of 30 ng/mL for both proteins was used.

As blood plasma displays a complex matrix, spike experiments in heparin plasma were performed to verify the aforementioned results. Comparable to the buffer results, the presence of PD-L1 did not influence the results of the PD-1 assay in any case. Additionally, PD-L2 and the combination of PD-L1 and PD-L2 did not decrease the recovery, only in very high concentrations of 60 ng/mL. PD-L1 samples were not affected by the presence of PD-1 as concentrations underneath 15 ng/mL did not disturb the measurements. However, PD-L2 concentrations did influence the PD-L1 results at concentrations of 0.23 ng/mL and above. Regarding the PD-L2 assay, PD-1 and PD-L1 affected the PD-L2 measurement only in the sample with the higher PD-L2 content (0.63 ng/mL): PD-L1 at concentrations higher than 3.75 ng/mL and PD-1 at concentrations higher than 0.94 ng/mL. In the presence of both PD-1 and PD-L1, the recovery was already affected at concentrations of 0.23 ng/mL. In the sample with a lower content of PD-L2 (0.38 ng/mL), influences of PD-1 were only observed at concentrations higher than 15 ng/mL, but not for PD-L1 (Figure 2, Figure S7).

The figure shows the recovery of the measured biomarker value in the presence of different concentrations of structurally similar proteins. For PD-1 (A) different concentrations of PD-L1, PD-L2 and the combination of both PD-L1 and PD-L2, for PD-L1 (B) different concentrations of PD-1, PD-L2 and the combination of both PD-1 and PD-L2 and finally for PD-L2 (C) different concentrations of PD-1, PD-L1 and the combination of both PD-1 and PD-L1 were used. Low and medium concentrations do not have a measurable effect on the assay performance. High concentrations, especially in combination of both proteins, can influence assay results, though. Dotted red lines frame the accepted recovery range within 80–120%.

### 3.6. Matrix Effects

**Figure 3:**
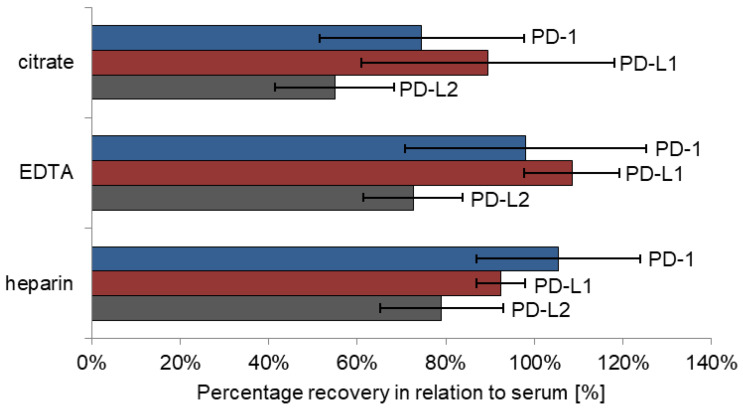
Matrix comparison recovery relative to serum concentration.

In comparison with serum samples, mean recoveries in heparin plasma (*n* = 5) ranged from 105.4% (CV: 18.5%) for PD-1, over 92.4% (CV: 5.5%) for PD-L1 to 79.0% (CV: 3.9%) for PD-L2 (Figure 3, Figure S8). Mean recoveries of EDTA plasma were comparable with serum levels for PD-1 (98.0%; CV: 27.3%) and PD-L1 (108.5%; CV: 10.8%), though PD-L2 levels were lower (72.6%; CV: 11.3%). Concerning citrate plasma, only mean recoveries of PD-L1 results stayed in the accepted ±20% range (89.5%; CV: 28.5%). Mean recoveries of PD-1 and PD-L2 showed considerable deviations to serum levels (PD-1: 74.6%; CV: 23.1% and PD-L2: 54.9%; CV: 13.4%).

Recoveries are calculated based on the measured serum value (serum value = 100%). The figure summarizes calculated means for all patients and matrices (heparin, EDTA and citrate plasma). Error bars display the corresponding coefficients of variation. Heparin values are identified as equal to serum, whereas citrate values are lower.

### 3.7. Healthy Subjects

Mean concentrations measured in a cohort of 136 healthy male individuals were 1.34 ng/mL (range 0.042–30.00 ng/mL) for PD-1, 0.012 ng/mL (range 0.0073–0.14 ng/mL) for PD-L1 and 0.96 ng/mL (0.064–29.011 ng/mL) for PD-L2 (Table 4).

## 4. Discussion

Programmed death-1 receptor PD-1 and its corresponding ligands PD-L1 and PD-L2 are key players in physiological immune tolerance and immune escape in cancer disease. As surface markers on tumor cells and diverse immune cells, but also as soluble markers circulating in the blood plasma and serum, they bear great potential to improve future oncoimmunology research and to be applied in monitoring immunotherapies in cancer patients. Applicability and clinical value of soluble PD markers have already been shown by studies on patients with diverse cancers, such as lung cancer, renal cell cancer and melanoma, receiving immune therapies [26,27].

For this purpose, high quality immunoassays are needed that enable the fast, reliable, robust and highly sensitive detection of these PD receptors and ligands in the blood plasma and serum. Therefore, the aim of the present work was the development of novel PD-1, PD-L1 and PD-L2 ELISAs and their comprehensive and thorough analytical validation as suggested by the European Medicine Agency (EMA) and a detailed description for biomarker validation of Andreasson et al. [22,23].

The methods were developed and optimized on a well-established chemiluminescent detection platform using commercially available, coordinated antibody pairs. The process was performed in a standardized way testing and defining appropriate antibody concentrations, buffers, type of plate, handling, standards, controls etc., resulting in robust and reliable detection methods. In a second step, the assay characteristics and performance were assessed by a comprehensive experimental setting. Good assay imprecision and dilution linearity, a wide measuring range, high sensitivity down to the pg/mL area, excellent selectivity of detection and a first application in healthy subjects have convincingly shown the fitness of the assays for use in oncoimmunologic research and clinical study settings.

Imprecision of all three assays was very good for the intra-assay comparison with CVs below 10% as well as for the inter-assay comparison with CVs below 20%. While during this early developmental stage all procedural steps were performed manually, further improvements are expected by future automatization of the processes.

Dilution linearity is especially important if very high results exceeding the measuring range are expected. Although the novel assays on the chemiluminescent platform were set up with a large dynamic range covering all of the pg/mL and ng/mL area, dilution may be relevant for extremely high values and to exclude high-dose-hooks effects. In our validation, experiments in assay diluent, artificial matrices and spiked samples showed recoveries close to the predefined values in all assays. Only in dilution of native heparin plasma were lower recoveries for PD-1 and PD-L2 observed in the first dilution step. As physiologically very low PD-L1 values are present in native samples, spike-in samples were used as starting material. Due to the wide measuring range and due to omitting any predilution steps of the samples before analysis, dilution remains of subordinate significance for assay performance.

However, analytical sensitivity of the assays indicated by LOD, LLOQ and ULOQ are important specifications for future clinical application, particularly to follow patients undergoing adjuvant therapies or monitoring minimal residual cancer disease. All three assays showed low to very low background signals (only 100 and 300 signal counts on the readout platform). Furthermore, low variation coefficients throughout the whole standard curve over different days proved a high reproducibility and robustness of the standards and the whole assay, enabling a broad quantification range over more than three orders of magnitude starting at below 10 pg/mL up to 30 ng/mL. This renders the assays highly sensitive tools for detecting also minimal amounts of the analytes of interest.

Analytical selectivity is an often neglected but highly important prerequisite for assay specificity. Consequently, it represents one of the key elements of the analytical validation in order to exclude any potential cross contamination of the measuring signal by analytes that are similar to the analyte of interest. To achieve high analytical selectivity, careful selection of highly specific antibodies is essential. As part of our study, a comprehensive set of experiments was carried out as especially the two ligands share a reasonable proportion of their protein structure. It has to be pointed out that all antibodies used in our assays proved high selectivity for their respective analytes in standard curve experiments. Influences on marker signals were only seen when non-physiological, extremely high concentrations of the related analytes were spiked into the samples investigated.

As a major influencing factor for analyte concentration, the choice of appropriate sample material and collection tubes has to be considered. As seen also for many other biomarkers, results for PD-1, PD-L1 and PD-L2 were quite comparable in serum and heparin plasma samples, respectively. In addition, levels of PD-1 and PD-L1 were similar in EDTA plasma samples; only PD-L2 levels were somewhat lower as compared with heparin plasma and serum. For all three analytes, recoveries measured in citrate plasma presents were considerably lower, which is partially explained by plasma volume reduction in the tubes due to prefilling with sodium citrate anticoagulant fluid. Nevertheless, it has to be emphasized that blood matrices should be maintained throughout studies to set optimal preconditions for comparable results.

As a summary of the analytical validation of three novel immunoassays for the quantification of PD-1, PD-L1 and PD-L2, all assays provide a robust basis for highly sensitive, specific and reliable measurement of the analytes in serum and plasma. They are based on commercially available coordinated antibody pairs which provide good quality and comparability, although batch stability has to be shown over time. Chemiluminescence detection has several advantages over color-based detection such as the precise quantification of minimal concentrations down to less than 10 pg/mL. Such excellent LLOQs are especially needed for PD-L1, as physiological marker levels are expected at these very low concentrations in blood serum and plasma. In addition, the broad dynamic measuring range with more than three orders of magnitude enables to capture low and high results at the same time and to avoid any predilution steps. This also facilitates the monitoring of individual marker courses over time, e.g., during or after immunotherapies in cancer patients as even large dynamic changes are easily detected. Recently, Goto et al. published a method using automated chemiluminescent immunoassays to determine concentrations of soluble PD-1 and PD-L1. The assays showed CVs lower than 10%, which can be explained by higher tested sample numbers and constant assay conditions by automatization. Obtained LODs and LOQs are comparable to the data calculated for the here-presented assays [28].

The investigation in a comprehensive set of healthy subjects proved that PD-1 and PD-L2 concentration can be measured with the newly established assays. The concentrations of PD-L1 were lower in the healthy population and often below the detection limit. Nevertheless, it is expected to detect PD-L1 concentrations in subjects with diseases as rising levels have been reported by increasing numbers of studies. Meanwhile, other publications on assays for the quantification of soluble PD-1 and PD-L1 have been released stressing the high interest in the field [26,27,28].

Although the assays have proven good analytical validity, their usage is so far limited to research applications and can be used for marker quantification in experimental and study settings. For in vitro diagnostics (IVD) qualification, further quality aspects as well as the potential influence of preanalytical factors have to be tested and fulfilled.

## 5. Conclusions

A comprehensive set of experiments proved good analytical validity for the newly developed, highly sensitive immunoassays detecting soluble PD-1, PD-L1 and PD-L2 in patient serum and plasma. They bear great potential to improve future oncoimmunology research and to be applied in monitoring immunotherapies in cancer patients.

## Figures and Tables

**Table 1:** Results for intra-assay imprecision experiments.

Assay	Sample	N	Mean Conc. (ng/mL)	CV (%)	Range (ng/mL)
PD-1	Sample I	11	0.58	7.8	0.52–0.67
	Sample II	12	0.29	6.5	0.23–0.31
	Sample III	15	0.06	6.4	0.05–0.07
PD-L1	Sample IV	12	0.43	3.8	0.40–0.45
	Sample V	12	0.03	4.2	0.02–0.03
	Sample VI	12	0.0038	7.1	0.0028–0.0048
PD-L2	Sample VII	11	7.60	4.5	7.46–8.43
	Sample VIII	12	1.21	10.0	1.00–1.51
	Sample IX	13	0.09	9.9	0.07–0.11

**Table 2:** Results for inter-assay imprecision experiments.

Assay	Sample	N	Mean Conc. (ng/mL)	CV (%)	Range (ng/mL)
PD-1	Sample X	12	0.17	12.7	0.13–0.22
	Sample XI	14	0.06	13.3	0.05–0.09
PD-L1	Sample XII	14	19.53	15.5	15.41–24.97
	Sample XIII	14	1.27	12.5	1.00–1.61
PD-L2	Sample XIV	12	3.40	13.2	2.50–3.94
	Sample XV	10	0.44	23.4	0.31–0.59

**Table 3:** Assay specifications including LOD, LLOQ and ULOQ.

Marker	LOD		LLOQ			ULOQ		
	N	Signal	N	Conc.	CV (%)	N	Conc.	CV (%)
PD-1	23	218	15	0.0073	9.1	16	30.0	17.5
PD-L1	30	226	5	0.0073	5.1	5	30.0	2.6
PD-L2	27	146	15	0.0073	16.1	16	30.0	6.0

**Table 4:** Soluble PD-1, PD-L1 and PD-L2 concentrations measured in healthy male subjects.

	PD-1 *	PD-L1 *	PD-L2 *
**Minimum**	0.042	0.0073	0.064
**1st Quartile**	0.13	0.0073	0.18
**Median**	0.23	0.0073	0.30
**Mean**	1.34	0.012	0.96
**3rd Quartile**	0.55	0.0095	0.57
**Maximum**	30.00	0.14	29.011

* Concentration in (ng/mL), *n* = 136 subjects.

## Data Availability

The data will be made available by the authors on request.

## Supplementary Materials

The following supporting information can be downloaded at: https://www.mdpi.com/article/10.3390/biomedicines10102405/s1, Figure S1: Antibody titration schema Standard plate and High bind plate; Figure S2: Dilution linearity in absolute concentrations (ng/mL) for PD-1, PD-L1 and PD-L2; Figure S3: Dilution linearity relative to undiluted value for different matrices; Figure S4: Dilution linearity relative changes to previous dilution (%) for PD-1, PD-L1 and PD-L2; Figure S5: Influence of the presence of PD-L1 and PD-L2 on the standard curve of the PD-1 assay and vice versa; Figure S6: Relative influence of the presence of PD-L1 and PD-L2 in different concentrations on the PD-1 assay and vice versa; Figure S7: Absolute influence of the presence of PD-L1 and PD-L2 in different concentrations on the PD-1 assay and vice versa; Figure S8: Absolute concentrations (ng/mL) of PD-1, PD-L1 and PD-L2 measured using different blood collection tubes. Table S1: Antibody concentrations in matrix titration experiment; Table S2: Comparison of Signal-to-Noise Ratios for the selected finally antibody concentrations on standard and high bind plate for all three assays; Table S3: Final assay conditions for PD-1, PD-L1 and PD-L2; Table S4: Results of the recovery of spiked PD-1, PD-L1 and PD-L2 concentrations in plasma samples applying a serial dilution.
