# Supplementary material for: High Quality Performance of Novel Immunoassays for the Sensitive Quantification of Soluble PD-1, PD-L1 and PD-L2 in Blood
Source: Biomedicines. 2022 Sep 26;10(10):2405. doi: 10.3390/biomedicines10102405 (PMC9598350; doi:10.3390/biomedicines10102405)
Supplement: Supplementary file 1 [file biomedicines-10-02405-s001.zip › biomedicines-1841620-supplementary.pdf]

biomedicines

MDPI

Article

High Quality Performance of Novel Immunoassays for the Sensitive Quantification of Soluble PD-1, PD-L1 and PD-L2 in Blood

Kimberly Krueger [1], Zsuzsanna Mayer [1], Miriam Gerckens [2], Stefan Boeck [2], Peter Luppa [3] and Stefan Holdenrieder [1,*]

[1] German Heart Center Munich, Clinics at the Technical University Munich, Institute of Laboratory Medicine, Munich Biomarker Research Center, 80636 Munich, Germany
[2] Department of Internal Medicine III, University Hospital Munich-Grosshadern, 81377 Munich, Germany
[3] Department of Clinical Chemistry, University Hospital of the Technical University Munich, 81675 Munich, Germany
* Correspondence: holdenrieder@dhm.mhn.de; Tel.: +0049-89-1218-1010

Abstract: Programmed death-1 receptor PD-1(CD279) and its corresponding ligands PD-L1(CD274, B7-H1) and PD-L2(CD273, B7-DC) play important roles in physiological immune tolerance and for immune escape in cancer disease. Hence establishment and analytical validation of a novel ELISA assays to measure soluble PD-1, PD-L1 and PD-L2 in blood samples according to high quality standards is required. Antibody pairs were used to establish novel highly sensitive ELISAs for all three markers on an open electrochemiluminescence Quickplex platform. Analytical validation comprised intra- and interassay imprecision, limit of quantification, dilution linearity, material comparison and analytical selectivity testing. The methods demonstrated a broad dynamic range and precise measurements down to the pg/ml range. The coefficient of variation (CV) during the intra-assay imprecision measurements with three patient pools did not exceed 10% for all three assays (PD-1: 6.4%, 6.5%, 7.8%, PD-L1: 7.1%, 4.2%, 6.8%; PD-L2: 4.5%, 10.0%, 9.9%). Dilution linearity experiments in both buffer and heparin plasma displayed good linearity. Selectivity was shown for each marker in titration cross-reactivity experiments up to concentrations of at least 15 ng/ml of these possibly confounding other markers. Soluble PD-1, PD-L1 and PD-L2 can be measured highly sensitively in serum and plasma and can safely be applied to clinical study settings.

Keywords: PD-1; PD-L1; PD-L2; analytical validation; ELISA

Supplementary Material

Table S1. Antibody concentrations in matrix titration experiment.

**Assay**	**Capture antibody concentration**	**Detection antibody concentration**
PD-1	**2µg/ml**<br>4µg/ml	100ng/ml<br>200ng/ml<br>**400ng/ml**
PD-L1	2µg/ml<br>**4µg/ml**	50ng/ml<br>**100ng/ml**<br>200ng/ml
PD-L2	**2µg/ml**<br>4µg/ml	100ng/ml<br>**200ng/ml**<br>500ng/ml

The bold concentrations are selected as the final antibody concentrations for the assays.

Table S2. Comparison of Signal-to-Noise Ratios for the selected finally antibody concentrations on standard and high bind plate for all three assays.

	**PD-1**		**PD-L1**		**PD-L2**	
	Standard plate	High bind plate	Standard plate	High bind plate	Standard plate	High bind plate
**STD1**	852,48	156,14	1092,03	352,59	821,87	226,13
**STD2**	222,94	50,85	566,96	165,53	189,00	55,56
**STD3**	62,80	15,10	175,31	49,78	46,14	14,25
**STD4**	15,16	4,33	42,59	13,53	12,14	4,27
**STD5**	4,43	1,81	11,31	4,10	3,51	1,73
**STD6**	1,81	1,17	3,95	1,77	1,48	1,13
**STD7**	1,26	1,09	1,70	1,20	1,02	1,04
**STD8**	1,00	1,00	1,00	1,02	1,00	1,00
**Mean increase (n-fold) Standard vs. High bind plate**	**3**		**3**		**2**	

STD: Calibration standard (as dilution series).

Table S3. Final assay conditions for PD-1, PD-L1 and PD-L2.

Assay	Plate type	Capture antibody concentration	Detection antibody concentration	Standard curve start concentration	Dilution	Diluent
**PD-1**	Standard	2µg/ml	400ng/ml	30ng/ml	1:4	MSD Diluent 2
**PD-L1**	Standard	4µg/ml	100ng/ml	30ng/ml	1:4	1%BSA in PBS
**PD-L2**	Standard	2µg/ml	200ng/ml	30ng/ml	1:4	MSD Diluent 2

Table S4. Results of the recovery of spiked PD-1, PD-L1 and PD-L2 concentrations in plasma samples applying a serial dilution.

	**PD-1**		**PD-L1**		**PD-L2**	
	Conc. in ng/ml	% to previous dilution	Conc. in ng/ml	% to previous dilution	Conc. in ng/ml	% to previous dilution
**N**	4		4		4	
**Neat**	0.046-0.085		<LOD		0.27-0.77	
**Spiked**	7.64-8.45		9.27-12.70		7.56-5.27	
**Dilution 1:2**	2.88-4.30	37-51	4.59-8.06	50-63	1.95-4.22	38-81
**Dilution 1:4**	1.10-2.57	38-60	2.75-3.57	60-65	1.26-2.21	41-64
**Dilution 1:8**	0.41-1.06	37-51	1.61-2.30	48-63	0.74-1.16	49-63
**Dilution 1:16**	0.17-0.59	41-60	0.93-1.35	56-59	0.24-0.81	32-110

<table>
<tr><th></th><th></th><th colspan="3">Capture AB conc. 2µg/ml</th><th colspan="3">Capture AB conc. 4µg/ml</th></tr>
<tr><th></th><th></th><th>1</th><th>2</th><th>3</th><th>4</th><th>5</th><th>6</th></tr>
<tr><td>A</td><td>STD1</td><td rowspan="8">Det. AB conc. 400ng/ml</td><td rowspan="8">Det. AB conc. 200ng/ml</td><td rowspan="8">Det. AB conc. 100ng/ml</td><td rowspan="8">Det. AB conc. 400ng/ml</td><td rowspan="8">Det. AB conc. 200ng/ml</td><td rowspan="8">Det. AB conc. 100ng/ml</td></tr>
<tr><td>B</td><td>STD2</td></tr>
<tr><td>C</td><td>STD3</td></tr>
<tr><td>D</td><td>STD4</td></tr>
<tr><td>E</td><td>STD5</td></tr>
<tr><td>F</td><td>STD6</td></tr>
<tr><td>G</td><td>STD7</td></tr>
<tr><td>H</td><td>STD8</td></tr>
</table>

AB: Antibody

Figure S1. Antibody titration schema Standard plate and High bind plate. The plate layout for the chessboard titration experiment is depicted in this figure. Two capture antibody concentrations were tested each against three detection antibody concentrations. The scheme was applied to both available plate types, standard and High bind.

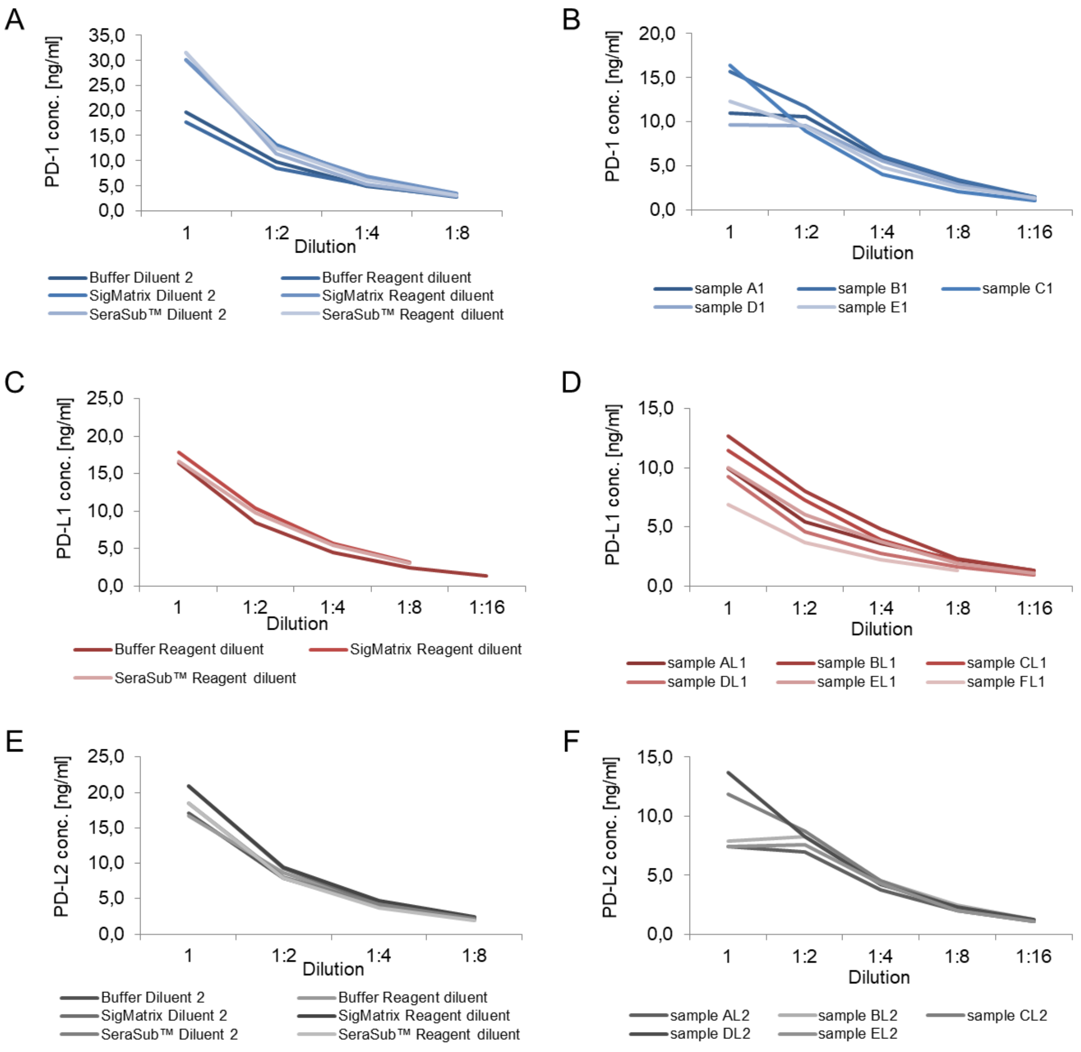

Figure S2. Dilution linearity in absolute concentrations (ng/ml) for PD-1, PD-L1 and PD-L2. The figures display the dilution linearity experiment results in different diluents and heparin plasma based on measured absolute concentrations. Graphs (**A**,**C**,**E**) show the results obtained in the buffer and artificial matrix experiments staring with PD-1, followed by PD-L1 and PD-L2. The second column containing graph (**B**,**D**,**F**) depict heparin plasma values in five to six participants.

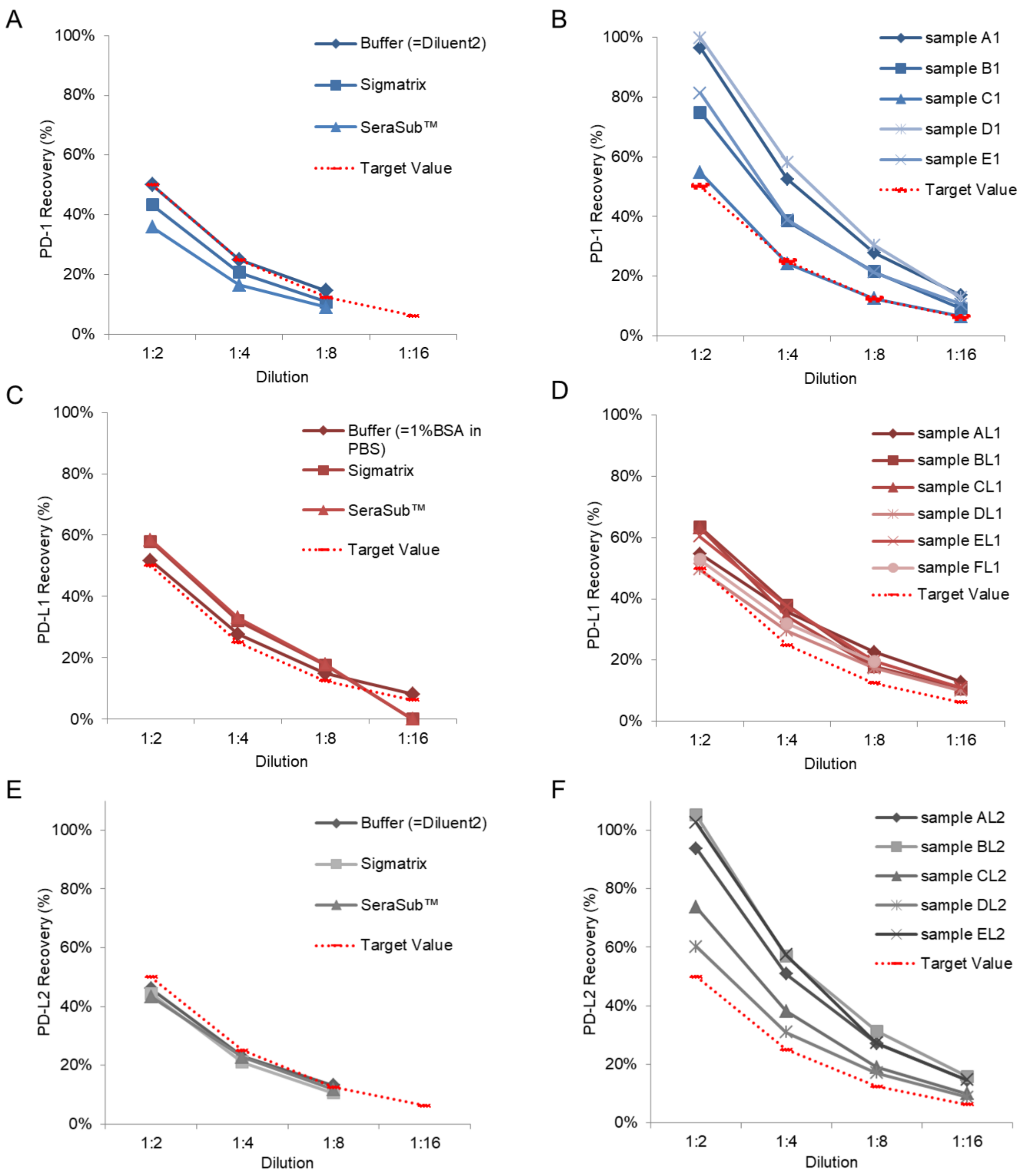

Figure S3. Dilution linearity relative to undiluted value for different matrices. Recoveries for PD-1, PD-L1 and PD-L2 are based on the undiluted sample value for buffer experiments in the left column (**A**,**C**,**E**) and for heparin samples in the right column (**B**,**D**,**F**). The dotted red line marks the target value starting with 50%. For PD-1 and PD-L2, the buffers show expected performance meanwhile the first dilution step of the samples results in higher variation. The biomarker PD-L1 convinces with recoveries close to the target value in all tested circumstances.

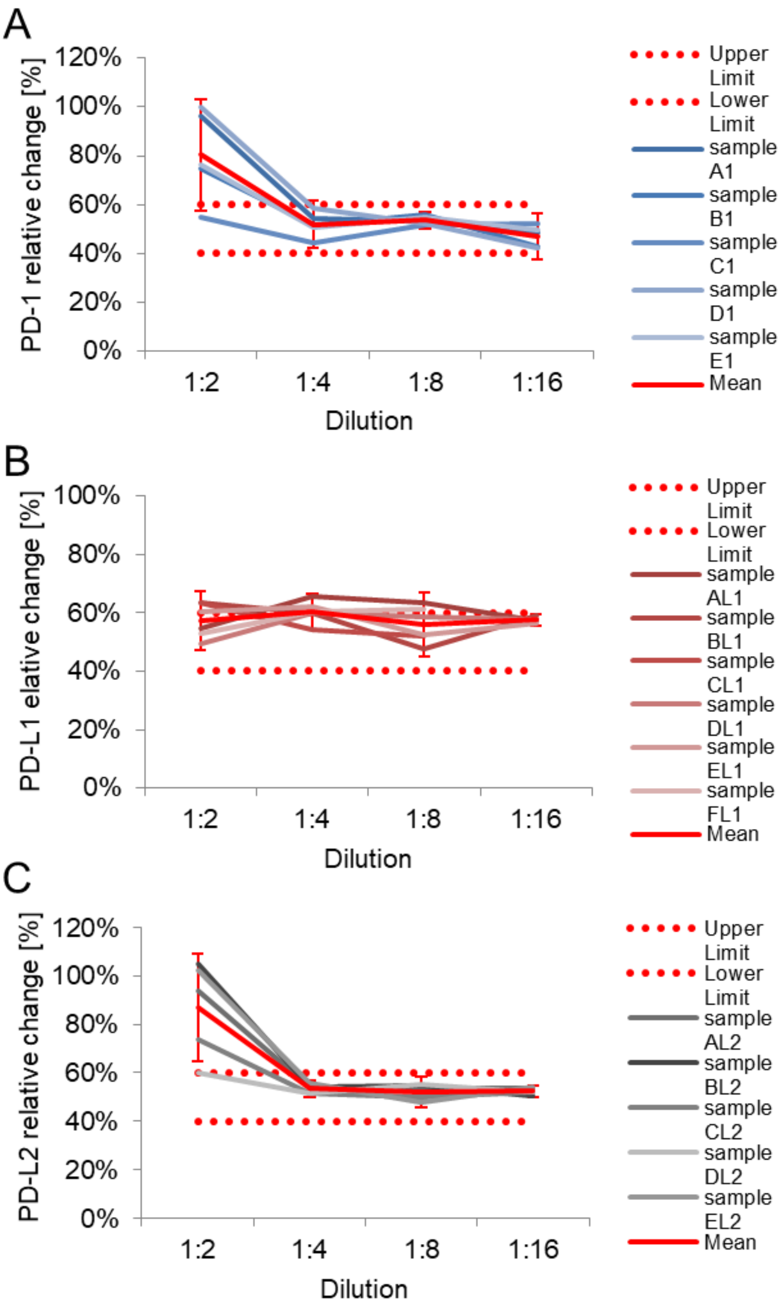

Figure S4. Dilution linearity relative changes to previous dilution (%) for PD-1, PD-L1 and PD-L2. The figure shows the relative change of biomarker value corresponding to the previous dilution in percent for PD-1 (**A**), PD-L1 (**B**) and PD-L2 (**C**). For each biomarker all patient courses are depicted separately and summarized as mean values (red line) including the error bars displaying corresponding CVs. The dotted red lines frame the target area ranging from 40 to 60%.

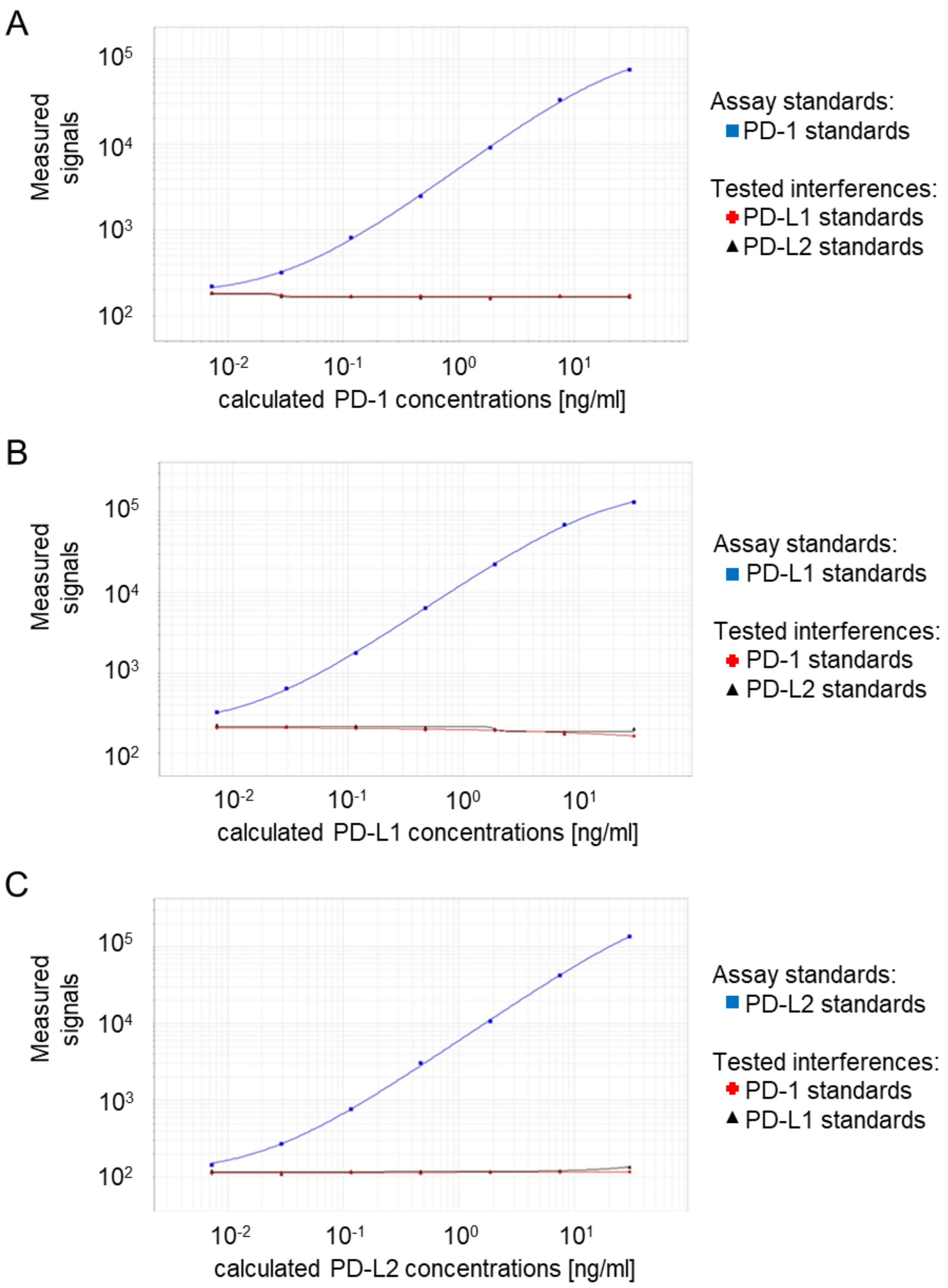

Figure S5. Influence of the presence of PD-L1 and PD-L2 on the standard curve of the PD-1 assay and vice versa. The graphs show the first part of the selectivity experiment, starting with PD-1 (**A**), followed by PD-L1 (**B**) and PD-L2 (**C**). For each assay, only the corresponding standard curve provides detectable signals whereas both of the other recombinant proteins only create signals equal to background signals.

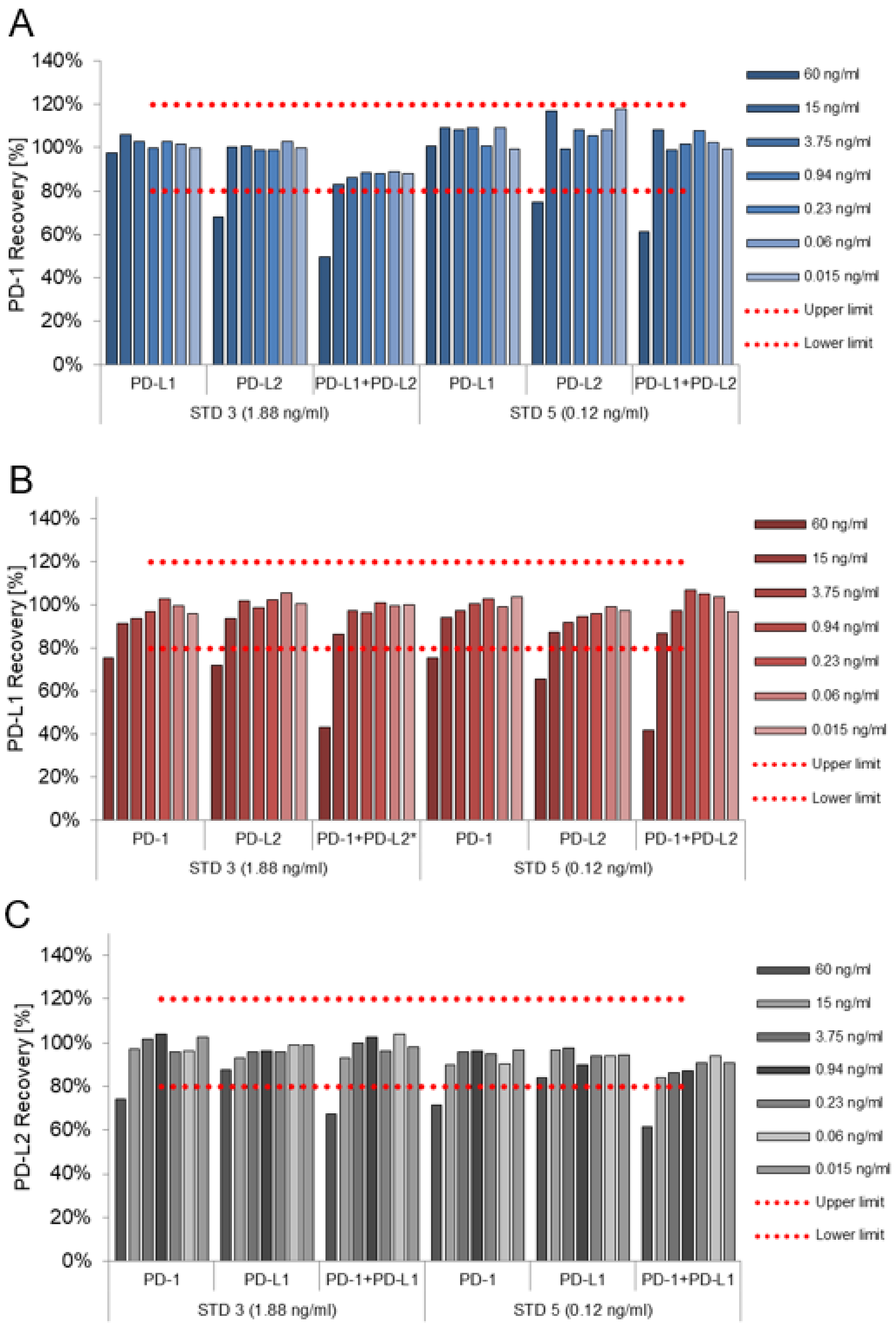

Figure S6. Relative influence of the presence of PD-L1 and PD-L2 in different concentrations on the PD-1 assay and vice versa. The figure displays the recovery of the measured target biomarker (e.g., PD-1) value in buffer meanwhile presence of different concentrations of structurally similar proteins (e.g., PD-L1 and PD-L2) (**A**). Subsequently, results for experiments with PD-L1 and PD-L2 as target biomarkers are shown (**B**,**C**). Dotted red lines mark the upper and lower acceptance criteria ranging from 80 to120%. No change on the target marker concentration can be obtained for low to medium concentrations of similar proteins. However, highest tested concentrations, especially in combination of both proteins, show an effect on assay performance.

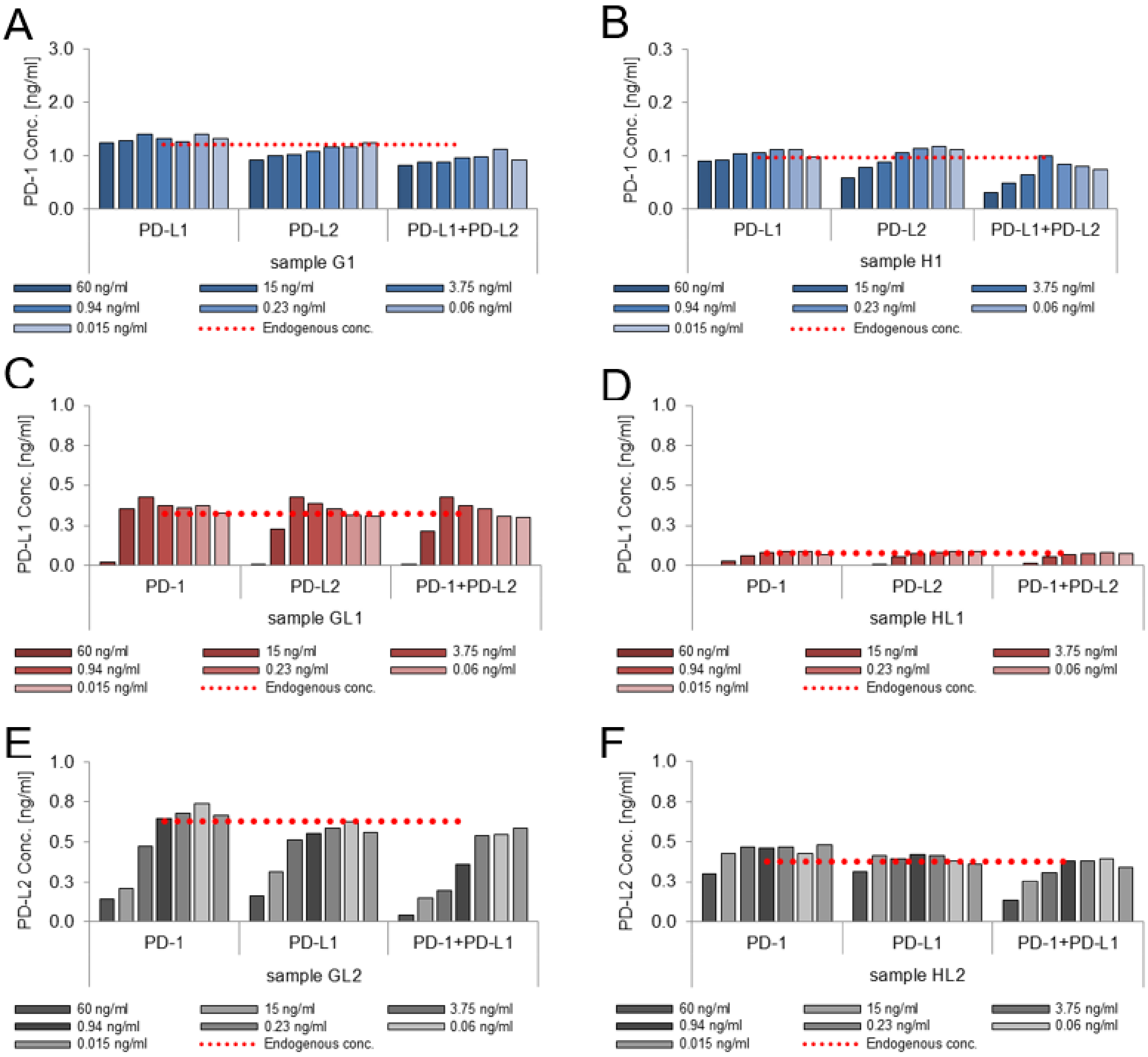

Figure S7. Absolute influence of the presence of PD-L1 and PD-L2 in different concentrations on the PD-1 assay and vice versa. The figure shows absolute concentration values obtained in heparin plasma obtained in the selectivity experimental set. Selectivity is tested in two different patient samples for PD-1 (**A**,**B**), PD-L1 (**C**,**D**) and PD-L2 (**E**,**F**) as the target marker. All measured concentrations for the different spike concentrations of corresponding potentially disturbing agents are displayed as bars. The dotted red line marks the endogenous target biomarker value. The closer the bars are to this red line, the less is the effect of the other proteins on the measurement.

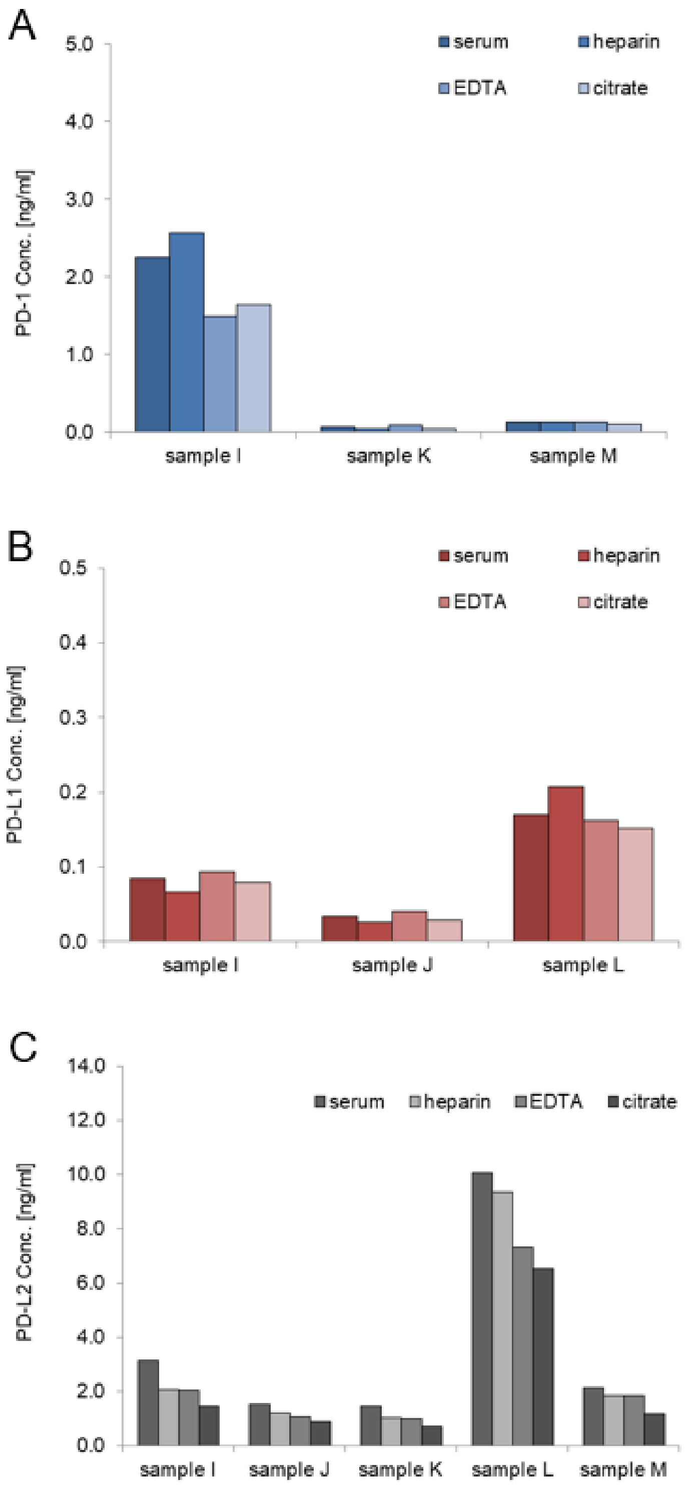

Figure S8. Absolute concentrations (ng/ml) of PD-1, PD-L1 and PD-L2 measured using different blood collection tubes. The graphs show absolute concentrations for the biomarkers PD-1 (**A**), PD-L1 (**B**) and PD-L2 (**C**) in the matrix comparison experiment. For up to five participants the obtained concentrations in four different blood sampling tubes, including serum, heparin, EDTA and citrate plasma, were compared.
